# Repair of Iatrogenic Furcal Perforation with Mineral Trioxide Aggregate: A Seven-Year Follow-up 

**DOI:** 10.22037/iej.v12i4.16888

**Published:** 2017

**Authors:** Jardel Camilo do Carmo Monteiro, Mateus Rodrigues Tonetto, Matheus Coêlho Bandeca, Alvaro Henrique Borges, José Cláudio Martins Segalla, Keren Cristina Fagundes Jordão-Basso, Cristian Fernando Sanchez-Puetate, Milton Carlos Kuga

**Affiliations:** a *Department of Restorative Dentistry, Araraquara School of Dentistry, Univ Estadual Paulista, Araraquara, SP, Brazil*;; b *Department of Postgraduate Program in Integrated Dental Science, University of Cuiaba-UNIC, Cuiabá, MT, Brazil;*; c *Department of Postgraduate Program in Dentistry, CEUMA University-UNICEUMA, São Luis, MA, Brazil*

**Keywords:** Endodontics, Furcation Perforation, Mineral Trioxide Aggregate, Root Canal Treatment, Root Perforation, Tooth Perforations

## Abstract

Teeth with furcal perforation present difficult resolution and dubious prognosis. Several materials have been proposed and calcium silicate-based cements such as mineral trioxide aggregate (MTA) are the most recommended. However, its long-term clinical behavior still remains poorly understood. The present study reports a clinical case of furcal perforation repair using Angelus MTA, with a 7-year follow-up. Patient sought treatment 2 months after iatrogenic accident. First lower right molar presented clinical signs such as fistula and bone loss between mesial and distal roots. Firstly, all root canals were treated and then furcal perforation was sealed with MTA Angelus and the dental crown was restored with composite resin. Radiographic evaluation was immediately performed to analyze the furcal perforation filling. After 7 years, a new clinical and imaging evaluation using periapical radiography and cone-beam computed tomography (CBCT) showed absence of clinical signs and symptoms, and alveolar bone reconstitution with periodontal space reduction. Angelus MTA presented good clinical behavior in the iatrogenic furcal perforation resolution based on long-term clinical evidence.

## Introduction

Furcal perforation is one of the most unpleasant and frequent accidents that can occur during endodontic treatment[1]. Burs with incompatible dimensions and/or inadequate direction during the pulp chamber ceiling removal and root canal location can contribute to this type of accident [2, 3].

Furcal and/or root perforation prognosis is unfavorable [4]. Dental extraction or perforation sealing using different materials such as endodontic or restorative cements are usually recommended and chosen based on prognosis [5-7]. The best clinical results were obtained using calcium hydroxide with different clinical strategies [3, 8].

However, large-sized furcal perforations do not respond favorably to calcium hydroxide, possibly due to its restricted physical and chemical properties[3, 8, 9]. Thus, other materials have been proposed to solve this problem, such as calcium silicate-based cements, which has demonstrated excellent biological and clinical results [9-13].

These cements have, currently, caused some scientific enthusiasm including new chemical modifications and/or associations with different vehicles in order to improve their clinical behavior, handling as well as biological properties [14, 15]. On the other hand, a long-term clinical assessment using this material is still unclear [16-18]. Mineral trioxide aggregate (MTA) is one of these calcium silicate cements that was introduced in 1990s and extensively studied to be used for perforation repairs, apexification, regenerative procedures, apexogenesis, pulpotomies, and pulp capping [19].

**Figure 1:**
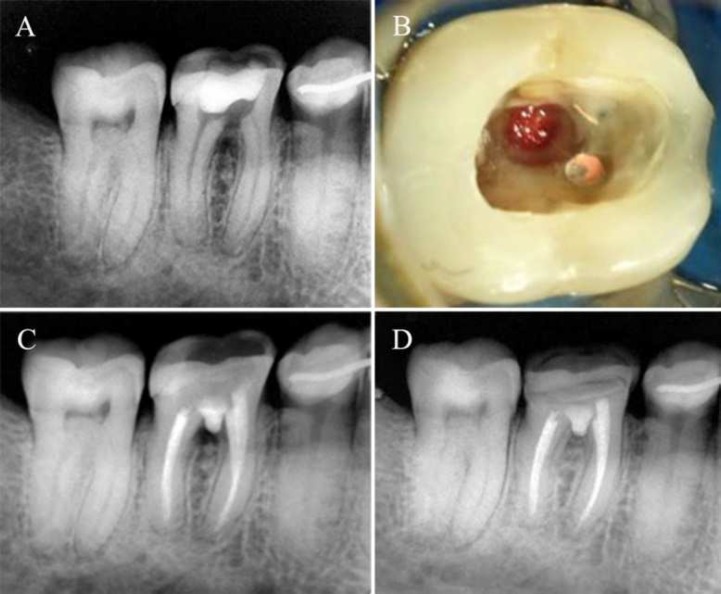
*A)* Initial radiographic image; *B)* Furcal perforation clinical image; *C)* Immediate radiographic image after perforation sealing; *D)* Radiographic image after 180 days follow-up

The present case report presents shows the clinical behavior of Angelus MTA after a perforation repair in a first mandibular molar using clinical, radiographic and bean-computed tomography evaluation after seven years.

## Case Report

A 40-year-old female patient sought clinical treatment due to unsuccessful endodontic treatment in right mandibular first molar. Clinical history revealed that the dentist was unable to locate the root canals and an iatrogenic accident occurred during pulp chamber access. Then, the endodontic treatment was interrupted, the pulp chamber was filled with calcium hydroxide paste (Ultracal XS; Ultradent, South Jordan, UT, USA), and the coronary access was temporarily restored using glass ionomer cement (Maxxion R; FGM, Joinville, SC, Brazil).

Initial clinical examination showed presence of fistula in gingival mucosa near to the radicular cervical region. Absolute isolation was carried out and then temporary restorative material and calcium hydroxide-based medication were removed. Pulp chamber was irrigated with saline solution, aspirated, and a visual inspection revealed a furcal perforation between the mesial and distal roots that presented measure similar to spherical bur #8 (Figure 1A). Periapical radiography revealed furcal perforation with significant communication to periodontal ligament (Figure 1B).

Furcal perforation was immediately filled with calcium hydroxide paste and sealed with glass ionomer cement. Then, the root cervical preparation was performed using ProTaper SX instrument (Dentsply Maillefer, Ballaigues, Switzerland). After obtaining glyde path was with a #10 K-file (Dentsply Maillefer, Ballaigues, Switzerland), real tooth length was obtained using an apex locator (ProPex; Dentsply Maillefer, Ballaigues, Switzerlands), and apical patency was performed with a Pathfile instrument (19/0.02) (Denstply Maillefer, Ballaigues, Switzerland). The root canals were instrumented up to F2 instrument (25/0.08). The root canals were irrigated with 2.5% sodium hypochlorite solution (Asfer, São Caetano do Sul, SP, BR) between instruments. Final irrigation was performed using 17% EDTA for 3 min and rinsed with 2.5% sodium hypochlorite solution [20].

**Figure 2:**
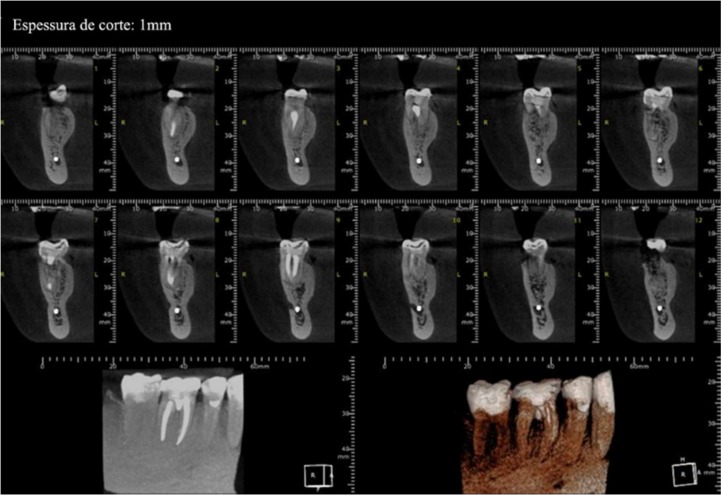
Cone-beam computed tomography images and periapical radiography after 7-year follow-up

Root canals were aspirated and dried with absorbent paper points, then endodontic obturation was performed with F2 gutta-percha (Dentsply Maillefer, Ballaigues, Switzerland) and epoxy resin-based sealer (AH-Plus; DeTrey/Dentsply, Konstanz, Germany) using the single-cone technique [21]. Endodontic sealer residues were cleansed using xylol in the pulp chamber, the glass ionomer cement protection was removed and the entire furcal perforation was irrigated with saline solution.

Perforation was then aspirated with specific endodontic tips (Cappilary Tips; Ultradent, South Jordan, UT, USA). MTA powder (Angelus, Londrina, PR, BR) was handled in a 1:1 ratio (powder and liquid), according to the manufacturer's instructions and inserted in the perforation zone. All the periodontal ligament was covered with MTA and confirmed by radiographic evaluation (Figure 1C). The area of perforation was approximately 2 mm and it was again protected using a glass ionomer cement and dental crown was restored using a self-etching adhesive system (Scotchbond Universal, 3M, SP, Brazil) and composite resin (Filtek Z350, 3M, SP, Brazil).

A new clinical and radiographic evaluation was performed after 180 days, showing bone repair in the inter-radicular region (Figure 1D) and the absence of clinical signs and symptoms. Seven years later, a new clinical evaluation using radiographic and cone beam-computed tomography was performed, revealing significant bone repair at furcation and the absence of clinical signs (Figure 2), showing success and clinical case resolution.

## Discussion

The prognosis of accidents involving pulp chamber floor anatomy is doubtful, and during decades, the only treatment was the tooth extraction. Calcium hydroxide was developed as an alternative treatment; however, due to its limited physical and chemical properties, some cases did not present good clinical results, especially for larger perforations [6, 8, 9].

Calcium silicate-based materials (MTA) have created new expectations in endodontic treatments, especially in cases that were considered lost in the past [3]. Currently, MTA is recommended in furcal and/or root perforation resolutions, among other indications, such as dental pulp conservative treatment, open root apices and periradicular surgery [12, 16, 17].

Due to the rather large perforation size in the present case, calcium hydroxide was avoided as partial or definitive treatment option, in accordance to Bryan *et al. *[3]. In addition, an immediate sealing with MTA was carried out since Holland *et al. *[8] have observed that medication with calcium hydroxide prior to MTA use did not favor local repair. Calcium hydroxide paste (Ultracal XS Ultradent products, Jordan, UT, USA) was initially placed in pulp chamber after perforation until the most proper treatment could be performed.

MTA is composed of SiO_2_, K_2_O, Al_2_O_3_, Na_2_O, Fe_2_O_3_, SO_3_, CaO, Bi_2_O_3_, MgO and CaO, KSO_4_, NaSO4 insoluble residues and crystalline silica. It presents favorable biological compatibility, favoring alkaline phosphatase activity, mineralized nodules formation and cell proliferation, as well as lower incidence of inflammatory chemical mediators favoring local tissue repair [11]. Although it promotes an immediate inflammatory reaction, a reduction in the number of inflammatory cells is observed after 60 days with significant periodontal space repair, under similar conditions to normal tissue [12].

MTA was selected as material of choice owing to its satisfactory biological properties. Radiographic and computed tomography analysis showed periodontal space regression confirming previously described microscopic findings [12]. Therefore, MTA also presented a satisfactory clinical result as a filling material in furcal perforations.

Various modifications in composition and/or handling techniques have been proposed to optimize the MTA use [5, 15, 22], however, the present study followed the manufacturer´s instructions maintaining the original composition. MTA modification in the composition and/or handling was avoided as long as no difficulties were found in the cement insertion at furcal perforation, although, studies have reported this difficulty [23, 24].

Long-term furcal perforation treatment clinical and imaging evaluation showed satisfactory clinical result after furcation repair with MTA which is a conservative and proper alternative for accident resolution and complications of endodontic origin.

## Conclusion

This clinical case concluded that mineral trioxide aggregate (MTA) presented a proper long-term clinical behavior for iatrogenic furcal perforation clinical and imaging evaluation confirmed by radiographic images and cone-beam computed tomography.
